# Beri Beri and Polished Rice

**Published:** 1913-11

**Authors:** 


Beri Beri and Polished Rice. Creighton, Wellman and Bass, Am. Soc. of Trop. Med., May, 1913, held that this was not a factor except negatively. Experiments on fowls and pigeons showed the same results from exclusive diet of potatoes and cereal breakfast foods.

